# The Effect of Metacognitive and Emotional Schemas on the Severity of Symptoms in Patients with Fibromyalgia

**DOI:** 10.3390/diagnostics16050696

**Published:** 2026-02-26

**Authors:** Mehmet Serhat Topaloğlu, Meltem Puşuroğlu

**Affiliations:** 1 Department of Physical Medicine and Rehabilitation, Faculty of Medicine, Recep Tayyip Erdoğan University, Rize 53100, Türkiye; 2 Department of Psychiatry, Faculty of Medicine, Recep Tayyip Erdoğan University, Rize 53100, Türkiye

**Keywords:** fibromyalgia, metacognition, emotions, schemas, chronic pain

## Abstract

**Background/Objectives:** The prevalence of mental disorders has increased in fibromyalgia (FM). Therefore, individuals’ pain perception, emotional schemas, and coping strategies are important. Our study aims to examine emotional schemas and metacognitive levels in FM. **Methods:** The study included 88 FM and 88 healthy controls who consecutively presented to the clinic. All participants completed the Leahy Emotional Schema Scale-II (LESS-II) and the Metacognition Questionnaire-30 (MCQ-30). Patients were also administered the Polysymptomatic Distress scale (PSD) and the Fibromyalgia Impact Questionnaire (FIQ). **Results:** The study included 88 FM patients and 88 controls. In the study, the LESS-II total score and MCQ-30 score were significantly higher in the patient group compared to the control group (*p* < 0.05). When examining the factors affecting FIQ severity, LESS-II scores and gender variables were found to be variables predicting FIQ values (*p* = 0.002, *p* = 0.002, respectively). When looking at the factors affecting the PSD score, the LESS-II score is a significant variable predicting the PSD score (*p* = 0.029). **Conclusions:** This study demonstrated that FM patients have higher levels of negative emotional schemas compared to healthy controls, and that emotional schemas are associated with both FM symptom severity and the impact of the disease on daily life. In particular, that LESS-II scores predict PSD scores and that LESS-II and gender variables predict FIQ scores suggests that symptom burden and functional effects in FM may be closely related to cognitive-emotional processes. The findings support the importance of considering emotional schemas in FM assessment and treatment approaches.

## 1. Introduction

Fibromyalgia (FM) is a disease characterized by symptoms such as widespread muscle and joint pain, fatigue, weakness, sleep disorders, and decreased cognitive capacity, arising from the interaction of biological, psychological, and environmental factors. It affects 2–3% of the world’s population. It often leads to disability due to its effects on the musculoskeletal system and its association with mental illness. The main symptom observed in patients is widespread and severe pain. Increased sensitivity is seen, especially in certain areas of the body. In addition, psychiatric symptoms such as fatigue, decreased sleep quality, difficulty concentrating, inability to sustain attention, and depressive mood accompany pain complaints in patients [1,2]. These cognitive complaints are often referred to in the literature as “fibrofog” and are defined as subjective impairments in attention, memory, and executive function. The etiology of the disease has not been fully elucidated. However, recent studies have shown an increase in the prevalence of mental disorders in FM patients. In particular, the prevalence of depressive disorder and anxiety disorder has increased in patients [3,4,5,6]. Symptoms such as pain and fatigue negatively affect patients’ problem-solving abilities. Patients, therefore, make up a group at higher risk for mental illness. The presence of mental illness also negatively affects FM symptoms. Studies have shown that mental illness negatively affects FM symptoms. Treating existing mental illness in patients also leads to improvement in FM symptoms. Therefore, it is important to recognize and treat accompanying mental illness in this patient group [7].

Metacognition is defined as being aware of one’s own self, recognizing and managing one’s feelings and self. Difficulties in areas such as developing problem-solving skills, emotional management, and expressing emotions are related to metacognition. Difficulty managing one’s own emotions leads to self-blame and negative feelings. According to the metacognitive model used in defining metacognition, an individual’s beliefs about their thoughts and cognitive processes play a decisive role in maintaining mental strain and continuing psychological symptoms in the face of stressful experiences. In this case, it leads to the development of mental illnesses such as depression. Studies have shown a relationship between metacognition and depression. Metacognition has also been linked to situations involving psychological distress. An individual’s metacognition and cognitive flexibility play an important role in their capacity to cope with situations where stress increases, such as chronic illnesses [8,9,10]. In this context, it is thought that fibrofog complaints, which are frequently reported in FM, may be related to metacognitive processes and beliefs about thought control. Emotional schemas are emotional teachings that a person develops, particularly from childhood onwards. Emotional schemas can play a role in regulating responses to events. Individuals’ coping mechanisms for stress and emotion management are related not only to cognitive methods but also to emotional schemas. When individuals are exposed to chronic stress, their coping methods are linked to their emotional schemas [11,12,13,14].

Chronic conditions such as FM increase patients’ stress levels. Emotional management, coping mechanisms, and emotional flexibility are important in dealing with chronic stress. The relationship between metacognition and emotional schemas and depression has been investigated. Individuals who struggle with emotion regulation and have more intense emotional schemas are more prone to mental illnesses such as depression. Research has shown that emotion-focused therapies are effective in patients [15]. However, in the context of FM, studies that address emotional schemas and metacognitive processes together are limited, and the relationship between these cognitive-emotional structures and symptom severity has not been sufficiently clarified yet. The relationship between mental illnesses, such as FM and depression, has been studied in the literature. However, emotion management, emotional schemas, and cognition are under-researched topics in this field. Emotional distress and stress increase in chronic conditions such as FM. Pain and other physical and mental symptoms negatively affect individuals’ quality of life [16,17]. Therefore, patients’ emotion management, cognitive flexibility, and stress management may be important in managing disease symptoms.

In addition, emotion- and cognition-focused approaches in patients’ treatment processes may lead to improvement in symptoms. However, research on this topic in the literature is limited. In the existing literature, FM has mostly been addressed through psychiatric comorbidities such as depression and anxiety; cognitive processes have generally been limited to catastrophizing or perceived stress variables. However, studies that examine the relationship between emotional schemas, which reflect the meanings individuals assign to their emotions and their patterns of coping with these emotions, and metacognitive processes, which encompass beliefs about thoughts and mental processes, with FM symptom severity and disease burden within the same model, are quite limited. In particular, examining these two structures together represents an important information gap in terms of revealing how symptoms in FM are affected not only by cognitive content but also by the way emotions are regulated and by metacognitive beliefs related to these processes. Our study will investigate the relationship between executive function and emotional schemas and FM symptoms in FM. The hypothesis that metacognition and emotional schemas are related to symptom severity was used as a starting point. In light of this information, this study aims to fill an important gap in the literature by examining the relationship between emotional schemas and metacognitive processes in FM patients and their disease symptoms. In addition to treatments targeting the musculoskeletal system, it is important to assess mental illnesses and include them in the treatment process when monitoring and treating patients.

## 2. Materials and Methods

### 2.1. Study Design and Participants

The study was conducted at the Physical Therapy and Rehabilitation Outpatient Clinic of Recep Tayyip Erdoğan University Education and Research Hospital. The study included 88 FM patients and 88 healthy controls who consecutively presented to the clinic. Similar studies in the literature were used as sources, and a power analysis was performed using G*Power version 3.1 (Düsseldorf, Germany). In the power analysis, the effect size was set at 0.3, the alpha value at 0.05, and the power of the study at 80%, resulting in a minimum sample size of 84 [18,19]. The diagnosis of FM was made in accordance with the updates proposed in the 2016 revision of the 2010/2011 diagnostic criteria of the American College of Rheumatology (ACR). Within this scope, the diagnosis was based on the combined evaluation of the Widespread Pain Index (WPI) and Symptom Severity Scale (SSS) scores, taking into account the criteria that the pain must have persisted for at least three months and cannot be better explained by another disease. Patients with chronic comorbidities and medication use, those unable to comply with the scales, those with a diagnosis of mental illness and taking medication, those with mental retardation, substance use disorders, traumatic brain injury that could invalidate self-reporting, cognitive sequelae of neurological conditions, and neuropsychological/neurocognitive disorders were excluded from the study. FM patients were evaluated by the same physical therapy and rehabilitation specialist. The severity of the disease symptoms was assessed using the Polysymptomatic Distress Scale (PSD), while its impact on daily life and functional status was assessed using the Fibromyalgia Impact Questionnaire (FIQ). Written and verbal information about the study was provided, and patients eligible to participate in the study were referred to a psychiatrist. Patients were evaluated by the same psychiatrist through a clinical interview based on DSM-5 (Diagnostic and Statistical Manual of Mental Disorders, Fifth Edition) criteria, administered the SCID-5 (Structured Clinical Interview for DSM-5), and patients without a mental illness diagnosis were included in the study. All participants were administered the Leahy Emotional Schema Scale-II (LESS-II) and the Metacognition Questionnaire-30 (MCQ-30). A healthy control group with similar sociodemographic characteristics to the patient group was also included in the study. The control group was screened for FM, chronic pain syndrome, and any medical or psychiatric condition that could cause functional limitations. In this context, participants underwent a brief clinical interview by the same psychiatrist, and the presence of psychiatric disorders was ruled out using the SCID-5. Scale forms were administered to participants who met the inclusion criteria. Written and verbal consent was obtained from patients in accordance with ethical principles at every stage of the study. Ethical committee approval for the study was obtained from the non-interventional ethics committee chair of Recep Tayyip Erdoğan University Faculty of Medicine (Ethics committee approval no: 2023/105).

### 2.2. Data Collection and Measurements

*Leahy Emotional Schemas Scale-II (LESS-II):* The original 50-item scale was revised, and a 28-item version was developed. The scale assesses emotional schemas and was developed within the framework of Leahy’s schema theories. It is a self-report scale. Higher scores are associated with increased use of emotional schemas. In our study, the total score of the scale was used [20]. It has been validated and verified in Turkish. It has valid internal consistency in the Turkish sample. In our research sample, the Cronbach’s alpha value of the scale was calculated as 0.74, and has sufficient internal consistency [21].

*Metacognition Questionnaire-30 (MCQ-30):* This was developed by Cartwright-Hatton and Wells. The validity and reliability of the scale in Turkish were established by Tosun and colleagues. The scale was found to have sufficient and valid internal consistency in Turkish society. In our research sample, the Cronbach’s alpha value of the scale was calculated as 0.82, indicating that it has valid internal consistency in our sample. High scores on the scale are interpreted as increased false metacognition. Cognitive self-consciousness (CS), Cognitive Confidence (CC), Uncontrollability and Danger (UD), Need for Control thoughts (NCT), and Positive Beliefs (PB) are the five sub-dimensions. In our study, both the total score of the scale and the scores of the subscales were used separately [22,23].

*Polysymptomatic distress scale (PSD):* This consists of the widespread pain index (WPI) and symptom severity scale (SSS) total. It is also known as the Fibromyalgia Symptom Scale (FS). In the first section, the location of the pain is determined. Localization such as the jaw, shoulder girdle, upper arm, forearm, hip, thigh, and leg is indicated, and 1 point is given for each section marked. This section forms the WPI. In the second section, fatigue, waking up feeling unrested in the morning, cognitive symptoms, headache, abdominal pain/cramps, and the presence of depression are assessed. A score between 0 and 12 is also obtained from this section. This section constitutes the SSS. To be diagnosed with FM, symptoms must have been present for at least 3 months, and the Widespread Pain Index must be ≥7 and the Symptom Severity Scale must be ≥5 points, or the Widespread Pain Index must be 4–6 and the Symptom Severity Scale must be ≥9 points [24,25].

*Fibromyalgia Impact Questionnaire (FIQ):* The FIQ is a 10-item self-administered instrument. The initial domain evaluates physical functioning, whereas the remaining domains assess weekly work loss, fatigue, pain, sleep disturbances, overall well-being, morning stiffness, psychological status, anxiety, and general health. A higher total score indicates a greater impact of the disease on FM [26].

### 2.3. Use of AI Tools

During the preparation of this study, we used ChatGPT-5.2 (OpenAI, San Francisco, CA, USA) and the ProWritingAid program to improve the language and readability and enhance the academic style of the article.

## 3. Statistics

The research data were analyzed using IBM SPSS Statistics version 29.0 (IBM Corp., Armonk, NY, USA). Descriptive statistics are presented as mean ± standard deviation, median (minimum- maximum) or frequency (%) values. The chi-square test was used to analyze categorical data. The normality of the data was examined using the Kolmogorov-–Smirnov Test, skewness and kurtosis values, and histogram visuals. For continuous variables found to follow a normal distribution, the difference between two independent groups was examined using the independent sample *t*-test, and for more than two groups, the one-way ANOVA test was used. The Bonferroni correction was applied for significant multiple comparisons. Cohen’s d effect size was calculated to determine the magnitude of the difference between groups. In interpreting the effect sizes, d = 0.20 was considered a small effect, d = 0.50 a medium effect, and d = 0.80 a large effect [18]. The relationship between variables was examined using the Pearson correlation test. Two separate regression models were created for FIQ and PSD score values in line with the research hypotheses. Stepwise multiple linear regression analysis was applied to determine the variables predicting FIQ and PSD scores. Age, gender (female), LESS-II total, MCQ-30 total score, and disease duration were included in the analysis as independent variables. The variables included in the regression analysis were evaluated stepwise based on those variables that contributed statistically significantly to the model, due to the limited preliminary evidence regarding the factors predicting FIQ and PSD scores and the exploratory nature of the study. The model assumptions were checked. The normality and distribution characteristics of the residuals were examined, and the possibility of multicollinearity was assessed through tolerance and VIF values. It was determined that the VIF values for all independent variables were below 2. Model fit and explanatory power were reported using R^2^ and adjusted R^2^ values. The statistical significance level was set at *p* < 0.05.

## 4. Results

A total of 176 participants were included in the study, comprising 88 patients and 88 healthy controls. When examining the mean age of the patient and control groups, the mean age of the patient group was 44.81 ± 9.08, while that of the control group was 42.32 ± 8.56. No statistically significant difference was found between the mean ages of the two groups (*p* = 0.063). A total of 11 patients were male (12.5%), 77 were female (87.5%), while 9 controls were male (10.2%) and 79 were female (89.8%). When examining the distribution of gender, education level, occupation, and marital status in the patient and control groups, no statistically significant differences were found between the groups (*p* = 0.635, *p* = 0.653, *p* = 0.371, *p* = 0.355, respectively) (Table 1).

When looking at the disease duration of the patient group, FIQ and PSD scores, the disease duration is a minimum of 6 and a maximum of 180 months, and the average is calculated as 65.5 ± 46.05 months. The average FIQ score is 64.69 ± 16.72, and the average PSD score is 19.76 ± 4 (Table 2).

When examining the relationship between the sociodemographic data of the patient group and their scale scores, no statistically significant relationship was found between age and FIQ (r = −0.03, *p* = 0.805) and PSD score (r = 0.11, *p* = 0.306) scores. When examining differences between groups by gender, it was found that women’s average FIQ scores (66.68 ± 14.79) were statistically significantly higher than men’s (50.74 ± 22.90) (*p* = 0.003). However, there was no significant difference in PSD scores between women (19.96 ± 3.84) and men (18.36 ± 4.97) (*p* = 0.217). No significant differences were found between groups in terms of marital status [FIQ (*p* = 0.337), PSD score (*p* = 0.640)], education level [FIQ (*p* = 0.714), PSD score (*p* = 0.294)], and occupational groups [FIQ (*p* = 0.438), PSD score (*p* = 0.221)] between groups (Table 3).

**Figure 1:**
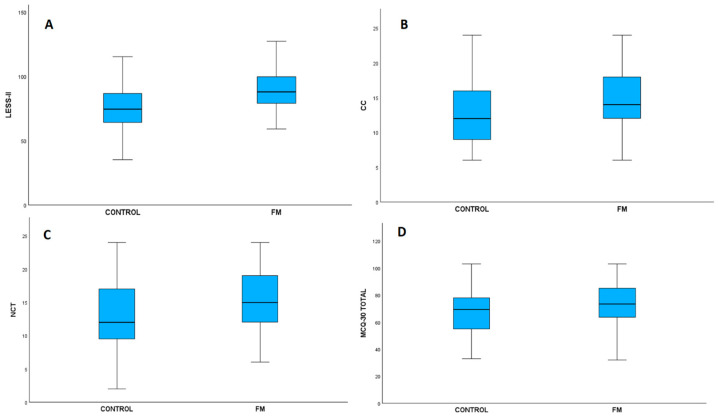
Boxplot graphs illustrating group comparisons between Control and FM participants. (**A**) LESS-II scores, (**B**) CC scores, (**C**) NCT scores, and (**D**) MCQ-30 Total scores. Each boxplot displays the median, interquartile range, and distribution of values for the respective parameter. Control and FM groups are shown side by side to highlight differences across measures. LESS-II: Leahy Emotional Schemas Scale-II, CC: Cognitive confidence, NCT: Need to control thoughts, MCQ-30: Metacognition Questionnaire-30, FM: Fibromyalgia.

The scale scores and subdimensions of the FM and control groups were compared. The LESS-II total score of the patient group (89.24 ± 15.52) is statistically higher than that of the control group (74.56 ± 16.91) (Figure 1A). The effect size was found to be high (Cohen’s d = 0.905). When looking at the MCQ-30 scale, the MCQ-30 total score was found to be significantly higher in the patient group (73.35 ± 15.14) compared to the control group (67.28 ± 16.04) (Figure 1D). The impact magnitude is low to moderate (Cohen’s d = 0.389). When examining the subscales of the MCQ-30 scale, it was determined that CC scores were significantly higher in the patient group (14.73 ± 4.81) compared to the control group (12.72 ± 4.82) (Figure 1B), and the effect size was moderate (Cohen’s d = 0.418). Similarly, in the NCT subscale, the patient group’s scores (15.39 ± 4.26) were significantly higher than those of the control group (13.02 ± 5.08). (Figure 1C) and the effect size was moderate (Cohen’s d = 0.504). In contrast, no statistically significant difference was found between the patient and control groups in the PB, UD, and CS subscales (Table 4).

The correlation between the scale scores of the patient group and the FIQ and PSD scores was examined. The LESS-II total score was moderately and positively correlated with the FIQ (r = 0.316, *p* = 0.003), and it had a small-to-medium positive correlation with the PSD score (r = 0.233, *p* = 0.029). When looking at the correlation with MCQ-30, a small-to-moderate and positive relationship was found between the MCQ-30 total score and the FIQ (r = 0.279, *p* = 0.008). Similarly, a positive relationship with a small effect size was found with PSD (r = 0.229, *p* = 0.032). Looking at the subscales, there is a moderate positive and significant correlation between UD and FIQ (r = 0.326, *p* = 0.002) and a small-to-moderate positive and significant correlation with the PSD score (r = 0.228, *p* = 0.032). There is a small-to-moderate positive correlation between NCT and FIQ (r = 0.238, *p* = 0.026), and between CS and FIQ (r = 0.237, *p* = 0.026). However, the relationships of both dimensions with the PSD score are not statistically significant (*p* > 0.05). No statistically significant relationship was found between the PB and CC subdimensions, and the FIQ and PSD scores (*p* > 0.05). To control for the possibility of Type I errors arising from multiple comparisons in correlation analyses, the Bonferroni correction was applied, and the corrected significance level was set at *p* < 0.006. After this adjustment, the relationships between the FIQ and the LESS-II total score (r = 0.316, *p* = 0.003), and between the FIQ and the UD subscale (r = 0.326, *p* = 0.002), remained statistically significant; other relationships were nominally significant but did not meet the adjusted threshold (Table 5).

In the stepwise multiple linear regression analysis conducted to evaluate the variables predicting the FIQ score, Model 2, which included the LESS-II and gender variables, was found to be statistically significant (F(2, 85) = 10.596, *p* < 0.001). The model explains 20% of the total variance in the dependent variable (R^2^ = 0.20, Adj. R^2^ = 0.18). When examining the model, LESS-II and gender variables were found to be predictors of FIQ scores (*p* = 0.002, *p* = 0.002, respectively). It has been determined that each unit increase in the LESS-II score is associated with a 0.33 unit increase in the FIQ value (B = 0.33, 95%Cl [0.13, 0.54]). When viewed in terms of the gender variable, being female was found to be associated with an average increase of 15.85 units in the FIQ variable score compared to males. (B = 15.85, 95%Cl [6.15, 25.54]) (Table 6).

In the stepwise multiple linear regression analysis conducted to predict the PSD score, only the LESS-II variable included in the model was found to be statistically significant (F(1, 86) = 4.95, *p* = 0.029). When examining the explanatory power of the model, it is seen that 5.5% of the variance in the PSD score is explained by this model (R^2^ = 0.055, Adj. R^2^ = 0.044). The LESS-II value is a significant variable predicting the PSD score dependent variable (*p* = 0.029). It has been determined that each unit increase in the LESS-II score is associated with a 0.06 unit increase in the PSD score. (B = 0.06, 95%Cl [0.06, 0.11]) (Table 7).

## 5. Discussion

This study investigated the relationship between emotional schemas and metacognition in individuals diagnosed with FM, and the severity of disease symptoms and disease management. According to the research results, the emotional schema scores and cognitive confidence, thought control, and metacognition scale total scores of the patient group are higher than those of the control group. An emotional schema consists of an individual’s thoughts about the emotions they feel, their beliefs about these emotions, and the coping methods they used to manage them. Metacognition is an individual’s awareness of their own mental processes [27,28,29]. The fact that higher scale scores were found in the patient group compared to the control group may suggest that patients use more maladaptive emotional schemas and experience difficulties in managing their emotions. The uncontrollability of thoughts and the individual’s lack of awareness of their own thought process may be associated with a greater symptom burden in patients. Research in the literature shows that mood disorders such as depression and anxiety are more common in patients with FM. This may be related to patients’ difficulties in managing their emotions [30]. In the correlation analysis results of the study, emotional schema total scores are positively related to both FIQ and PSD scores, while metacognitive total scores are positively related to FIQ values. In light of these results, it can be considered that maladaptive emotional schemas and difficulties in mental control are related to the severity of the disease symptoms. However, after applying the Bonferroni correction for multiple comparisons, the associations with PSD lost statistical significance; only the positive association between FIQ and the LESS-II score as well as the MCQ-30 UD subscale remained statistically significant. This situation suggests that some of the relationships initially observed may have been due to the multiple testing effect, however, it indicates that maladaptive emotional schemas and uncontrollable thoughts, in particular, show a stronger and more consistent relationship with the level of disease impact. However, the fact that the relationships identified with PSD lose their significance in adjusted analyses suggests that this variable’s relationship with disease severity may be weaker or more indirect and should be reevaluated in larger samples. In our study, FIQ and PSD score values were examined separately. Differences in the clinical interpretation of both scales are therefore important. The FIQ values primarily assess the overall impact of FM on the patient’s daily life and the level of psychological distress, while the PSD scale primarily assesses the severity of the disease and is also used in the diagnosis of FM [25,31]. In this case, the association of metacognition with FIQ suggests that metacognition and mental awareness may influence symptom perception, quality of life, and disease burden. In regression analyses, similar to correlation, LESS-II score was found to be a predictor for both FIQ and PSD scores. However, MCQ-30 score lost its significance in these models for FIQ and PSD scores. This situation may suggest that emotions and the meanings attached to them may be higher in FM patients than mental processes. Although the explanatory power of the models is limited, when evaluated in the context of the literature, it is thought that the observed relationship may have value in developing hypotheses for clinical application. Additionally, female gender was found to be a significant predictor for FIQ scores. Furthermore, the average FIQ scores for women were higher than those of the healthy control group. There was a difference in the gender distribution between women and men in our research sample. Although this situation reduces statistical power, it can be interpreted as meaning that emotional symptoms may be more common in women clinically diagnosed with FM than in men. Additionally, FM is more common in women than in men. Emotional distress may be more common in women due to factors such as societal roles attributed to women and the effects of hormones [17,32]. Our study did not examine emotional schemas and metacognition sociodemographic factors in detail. However, the literature indicates that women are a group at higher risk for mental illness. Women may experience greater difficulty coping with emotional stress and managing their emotions. This may be one reason why biopsychosocial diseases such as FM are more common in women. However, at this point, psychological and emotional factors specific to women are complex. Emotional intelligence and emotional awareness yield different results in women [30,33].

Our research findings generally suggest that emotional schemas are associated with FM symptoms. Research in this area is limited in the literature. A study by Paschali and colleagues showed that an increase in emotional schemas in FM patients was associated with increased pain perception and increased symptom severity. It was demonstrated that increased catastrophizing schemas in patients played a mediating role in symptom severity [16]. Similarly, another study found that early maladaptive schemas increase disease severity in FM patients. It has been reported that patients have more negative emotional schemas, which may negatively affect the prognosis of the disease [34]. In FM patients, an increase in the frequency of negative emotions and difficulty managing emotions may be observed. The long-term chronic course of the disease can also be exhausting for patients. Patients may find it difficult to express their feelings. In some cases, emotional stress and the severity of disease symptoms can be intertwined. This situation can increase the severity of disease symptoms. In particular, the burden of chronic illness can lead to increased emotional stress in patients. Recognizing emotions and emotion-focused therapies can contribute positively to the prognosis of the disease [35,36]. Studies investigating the relationship between emotional schemas and FM symptoms are limited in the literature. However, emotional schemas play an important role in the development of mental disorders such as depression and anxiety [37]. Emotional distress in patients can lead to the development of mental illness. In this case, coping with emotional stress can become more difficult in chronic diseases such as FM. Consistent with this information, our research shows that emotional schemas are higher in the patient group and may be related to symptom severity. Metacognition, like emotional schemas, may also play a role in the development of mental illnesses. Mindfulness, which is awareness of oneself and one’s emotions, plays a role in helping individuals cope with emotional stress through the management of their own thoughts. The development of problem-solving skills is also related to the development of the patient’s metacognition [38]. In our study, CC and the NCT, which are sub-dimensions of the MCQ-30, were high in the patient group. A review of the literature suggests that maladaptive metacognitive beliefs are associated with both emotional and physical symptoms [39]. A study conducted by Romeo and colleagues found a relationship between metacognition and pain scores in FM patients. Furthermore, it was demonstrated that depression and anxiety mediate the relationship between metacognition and pain in these patients [19]. Similarly, another study investigated the relationship between metacognition, anger, and pain symptom severity. According to the study results, maladaptive metacognitive beliefs are positively associated with greater pain symptom severity in patients with fibromyalgia [40]. Negative cognitive processes in patients may exacerbate pain, leading to an increase in perceived pain. Experiencing persistent and chronic pain can cause both emotional and physical distress. Therefore, chronic pain and emotional distress can be associated with negative cognitive processes [41,42].

Our study has some limitations. It is a single-center study, and all participants were included from the same region. Therefore, the study results cannot be generalized. The explanatory power of the models we established in the study is low. This indicates that there are additional factors influencing FM symptoms. However, our study did not examine other factors that could affect symptom severity, such as family support, socioeconomic status, perceived stress, exercise, and diet. This is a significant limitation. Studies that investigate multiple factors together with larger samples will contribute to the literature. Furthermore, the use of FIQ instead of Fibromyalgia Impact Questionnaire Revised (FIQR) may limit direct score comparisons with some recent studies reporting FIQR, however, FIQ is frequently used as a valid and reliable scale for assessing the impact of FM. Our study is cross-sectional and does not provide strong results in terms of causality. However, when evaluated in a clinical context, the findings are considered to contribute to the literature and may be clinically noteworthy. Furthermore, women constitute the majority of our research sample. This is a limitation from a statistical perspective. However, it is valuable to conduct multidisciplinary research on a topic that has been little studied in the literature in a specific patient group. It will contribute to clinical knowledge in the literature and shed light on further research.

## 6. Conclusions

Our research has shown that emotional schemas may be related to disease symptoms. Our findings support the notion that FM is not a clinical condition limited solely to the musculoskeletal system; rather, it is a biopsychosocial disease closely related to emotional, cognitive, and psychological processes. Emotion-focused research in FM patients will offer a new perspective on the etiology and treatment of the disease. Developing emotional stress management and emotion-focused coping methods is important in FM. Identifying mental illnesses in the follow-up and treatment of patients will contribute positively to the prognosis. Negative emotional schemas and reduced awareness can make it difficult for patients to recognize, express, and regulate their emotions. As a result, difficulty expressing emotions can lead to emotional stress being expressed as physical pain. Furthermore, the presence of early negative schemas can deepen difficulties in expressing emotions, leading to the chronicity of symptoms. Negative early childhood schemas can also make it difficult to express emotions. This study examines the relationship between emotional schemas and metacognitive processes in the context of disease symptom severity and disease management. In this respect, it aims to contribute to an area that has been relatively understudied in the literature. The FM literature often focuses on core symptoms such as pain, fatigue, and functional limitations; furthermore, the disease is mostly addressed within the framework of psychiatric comorbidities such as depression and anxiety. The role of emotional schemas in the perception and maintenance of symptoms has been evaluated in a limited number of studies. This study contributes to understanding FM based on emotion-focused and emotional schemas by revealing the relationship between emotional schemas and FM symptom severity, as well as their association with coping processes. Treatments targeting the musculoskeletal system are precious in FM therapy. However, neglecting mental illnesses prevents patients from achieving full well-being. Therefore, it is important to follow up with patients using a multidisciplinary approach. Evaluating the potential protective and therapeutic effects of interventions aimed at enhancing emotion regulation skills, emotional awareness, and emotional expression on FM symptoms could provide significant contributions to clinical practice.

## Figures and Tables

**Table 1:** Sociodemographic data of the patient and control groups.

		Control (*n* = 88)	FM (*n* = 88)	*p*
Age (years)	Mean ± SD	42.32 ± 8.56	44.81 ± 9.08	0.063 ᵃ
Gender	Male, *n* (%)	9 (10.2)	11 (12.5)	0.635 ᵇ
	Female, *n* (%)	79 (89.8)	77 (87.5)	
Education level	Primary school, *n* (%)	21 (23.9)	23 (26.1)	0.653 ᵇ
	High school, *n* (%)	45 (51.1)	39 (44.3)	
	University, *n* (%)	22 (25.0)	26 (29.5)	
Occupation	Civil servant, *n* (%)	17 (19.3)	11 (12.5)	0.371 ᵇ
	Worker, *n* (%)	10 (11.4)	14 (15.9)	
	Unemployed, *n* (%)	61 (69.3)	63 (71.6)	
Marital status	Single, *n* (%)	21 (23.9)	16 (18.2)	0.355 ᵇ
	Married, *n* (%)	67 (76.1)	72 (81.8)	

ᵃ Independent samples *t*-test, ᵇ Pearson chi-square test, *p* < 0.05, FM: Fibromyalgia, SD: Standard deviation.

**Table 2:** Descriptive statistics of the FM’s disease duration, FIQ and PSD scores (*n* = 88).

	Minimum	Maximum	Mean ± SD
Disease duration (months)	6	180	65.5 ± 46.05
FIQ	23.6	91.1	64.69 ± 16.72
PSD	12	30	19.76 ± 4

FIQ: Fibromyalgia Impact Questionnaire, PSD: Polysymptomatic Distress scale, SD: Standard Deviation. The duration of illness is presented as the total time in months after diagnosis.

**Table 3:** Analysis of the FIQ and PSD scores of the FM group according to sociodemographic data.

		FIQ		PSD	
		r	*p*	r	*p*
Age		−0.03	0.805 ^a^	0.11	0.306 ^a^
		mean ± SD	*p*	mean ± SD	*p*
Gender	Female	66.68 ± 14.79	0.003 *^,b^	19.96 ± 3.84	0.217 ^b^
	Male	50.74 ± 22.90		18.36 ± 4.97	
Marital Status	Married	63.88 ± 16.73	0.337 ^b^	19.39 ± 3.77	0.640 ^b^
	Single	68.34 ± 16.69		21.44 ± 4.68	
Education	Primary school	64.89 ± 18.16	0.714 ^c^	20.57 ± 3.79	0.294 ^c^
	High school	66.02 ± 14.83		19.92 ± 4.33	
	University	62.52 ± 18.44		18.81 ± 3.60	
Occupation	Civil servant	58.77 ± 16.16	0.438 ^c^	17.82 ± 3.60	0.221 ^c^
	Worker	66.80 ± 19.23		19.79 ± 3.70	
	Unemployed	65.26 ± 16.27		20.10 ± 4.09	

^a^ Pearson correlation, ^b^ Independent Sample *t*-test, ^c^ One Way ANOVA, *p* < 0.05, * *p* < 0.05 Significant results are highlighted in bold. SD: Standard Deviation, PSD: Polysymptomatic Distress Scale, FIQ: Fibromyalgia Impact Questionnaire, FM: Fibromyalgia. According to the results of the one-way ANOVA, there was no statistically significant difference between the groups (*p* > 0.05). Therefore, no post-hoc analysis was performed.

**Table 4:** Comparison of LESS-II, MCQ-30, and subscale scores between the FM and control groups.

	FM (Mean ± SD)	Control (Mean ± SD)	*p*	Cohen’s d (95%CI)
LESS-II TOTAL	89.24 ± 15.52	74.56 ± 16.91	<0.001 **	0.905 (0.593–1.214)
PB	11.98 ± 4.36	11.61 ± 4.17	0.573	0.085 (0.211–0.381)
UD	13.94 ± 4.17	13.23 ± 3.59	0.213	0.189 (0.108–0.484)
CC	14.73 ± 4.81	12.72 ± 4.82	0.006 *	0.418 (0.118–0.716)
NCT	15.39 ± 4.26	13.02 ± 5.08	0.001 *	0.504 (0.203–0.804)
CS	17.33 ± 4.01	16.70 ± 3.74	0.292	0.159 (0.137–0.455)
MCQ-30 TOTAL	73.35 ± 15.14	67.28 ± 16.04	0.011 *	0.389 (0.090–0.687)

Independent samples *t*-tests, *p* < 0.05, Descriptive statistics are presented as “Mean ± SD.” * *p* < 0.05, ** *p* < 0.001 Significant results are highlighted in bold. Cohen’s d was calculated by dividing the difference between the means of the patient and control groups by the combined standard deviation. LESS-II: Leahy Emotional Schemas Scale-II, PB: Positive beliefs, UD: Uncontrollability and danger, CC: Cognitive confidence, NCT: Need to control thoughts, CS: Cognitive self-consciousness, MCQ-30: Metacognition Questionnaire-30, FM: Fibromyalgia.

**Table 5:** Correlation of FIQ and PSD scores in the FM group with LESS-II, MCQ-30, and subscale scores.

		FIQ	PSD
LESS-II TOTAL	r	0.316	0.233
	*p*	0.003 *	0.029
PB	r	0.970	0.191
	*p*	0.370	0.075
UD	r	0.326	0.228
	*p*	0.002 *	0.032
CC	r	0.107	0.155
	*p*	0.320	0.150
NCT	r	0.238	0.147
	*p*	0.026	0.172
CS	r	0.237	0.088
	*p*	0.026	0.417
MCQ-30 TOTAL	r	0.279	0.229
	*p*	0.008	0.032

Pearson correlation, *p* < 0.05 * Adj. Bonferroni *p* < 0.006. Significant results are highlighted in bold. FIQ: Fibromyalgia Impact Questionnaire, PSD: Polysymptomatic Distress Scale, LESS-II: Leahy Emotional Schemas Scale-II, PB: Positive beliefs, UD: uncontrollability and danger, CC: cognitive confidence, NCT: Need to control thoughts, CS: Cognitive self-consciousness, MCQ-30: Metacognition Questionnaire-30, FM: Fibromyalgia.

**Table 6:** Stepwise regression model for determining variables affecting the FIQ score.

	B (95%CI)	SE	β	t	*p*
constant	20.59 (0.01, 41.19)	10.35	—	1.98	0.050
LESS-II	0.33 (0.13, 0.54)	0.10	0.314	3.24	0.002 *
Gender (Female)	15.85 (6.15, 25.54)	4.87	0.315	3.25	0.002 *

R^2^ = 0.20, Adj. R^2^ = 0.18, F(2, 85) = 10.596, Model 2 *p* < 0.001, Durbin–Watson: 1.808. B: Unstandardized Coefficient, β: Standardized Coefficient, SE: Standard estimation, CI: Confidence Interval, LESS-II: Leahy Emotional Schemas Scale-II, FIQ: Fibromyalgia Impact Questionnaire. * *p* < 0.05 Significant results are highlighted in bold.

**Table 7:** Stepwise regression model for ıdentifying variables affecting the PSD score.

	B (95%CI)	SE	β	t	*p*
Constant	14.39 (9.52, 19.25)	2.45	—	5.88	<0.001
LESS-II	0.06 (0.06, 0.11)	0.03	0.233	2.22	0.029 *

R^2^ = 0.055, Adj. R^2^ = 0.044, F (1, 86) = 4.95, Model 1 *p* = 0.029, Durbin–Watson = 1.794. B: Unstandardized Coefficient, β: Standardized Coefficient, SE: Standard estimation, CI: Confidence Interval, PSD: Polysymptomatic Distress Scale, LESS-II: Leahy Emotional Schemas Scale-II. * *p* < 0.05 Significant results are highlighted in bold.

## Data Availability

The datasets used and/or analyzed in the present study are available from the corresponding author upon reasonable request.
